# High-yield α-humulene production in *Yarrowia lipolytica* from waste cooking oil based on transcriptome analysis and metabolic engineering

**DOI:** 10.1186/s12934-022-01986-z

**Published:** 2022-12-24

**Authors:** Qi Guo, Qian-Qian Peng, Ying-Ying Chen, Ping Song, Xiao-Jun Ji, He Huang, Tian-Qiong Shi

**Affiliations:** 1 College of Biotechnology and Pharmaceutical Engineering, Nanjing Tech University, No. 30 South Puzhu Road, Nanjing, 211816 People’s Republic of China; 2 School of Food Science and Pharmaceutical Engineering, Nanjing Normal University, 2 Xuelin Road, Qixia District, Nanjing, 210046 People’s Republic of China; 3 College of Pharmaceutical Sciences, Nanjing Tech University, No. 30 South Puzhu Road, Nanjing, 211816 People’s Republic of China

**Keywords:** *Y. lipolytica*, Transcriptome analysis, Waste cooking oil, α-humulene

## Abstract

**Background:**

α-Humulene is an important biologically active sesquiterpene, whose heterologous production in microorganisms is a promising alternative biotechnological process to plant extraction and chemical synthesis. In addition, the reduction of production expenses is also an extremely critical factor in the sustainable and industrial production of α-humulene. In order to meet the requirements of industrialization, finding renewable substitute feedstocks such as low cost or waste substrates for terpenoids production remains an area of active research.

**Results:**

In this study, we investigated the feasibility of peroxisome-engineering strain to utilize waste cooking oil (WCO) for high production of α-humulene while reducing the cost. Subsequently, transcriptome analysis revealed differences in gene expression levels with different carbon sources. The results showed that single or combination regulations of target genes identified by transcriptome were effective to enhance the α-humulene titer. Finally, the engineered strain could produce 5.9 g/L α‐humulene in a 5‐L bioreactor.

**Conclusion:**

To the best of our knowledge, this is the first report that converted WCO to α-humulene in peroxisome-engineering strain. These findings provide valuable insights into the high-level production of α-humulene in *Y. lipolytica* and its utilization in WCO bioconversion.

**Graphical Abstract:**

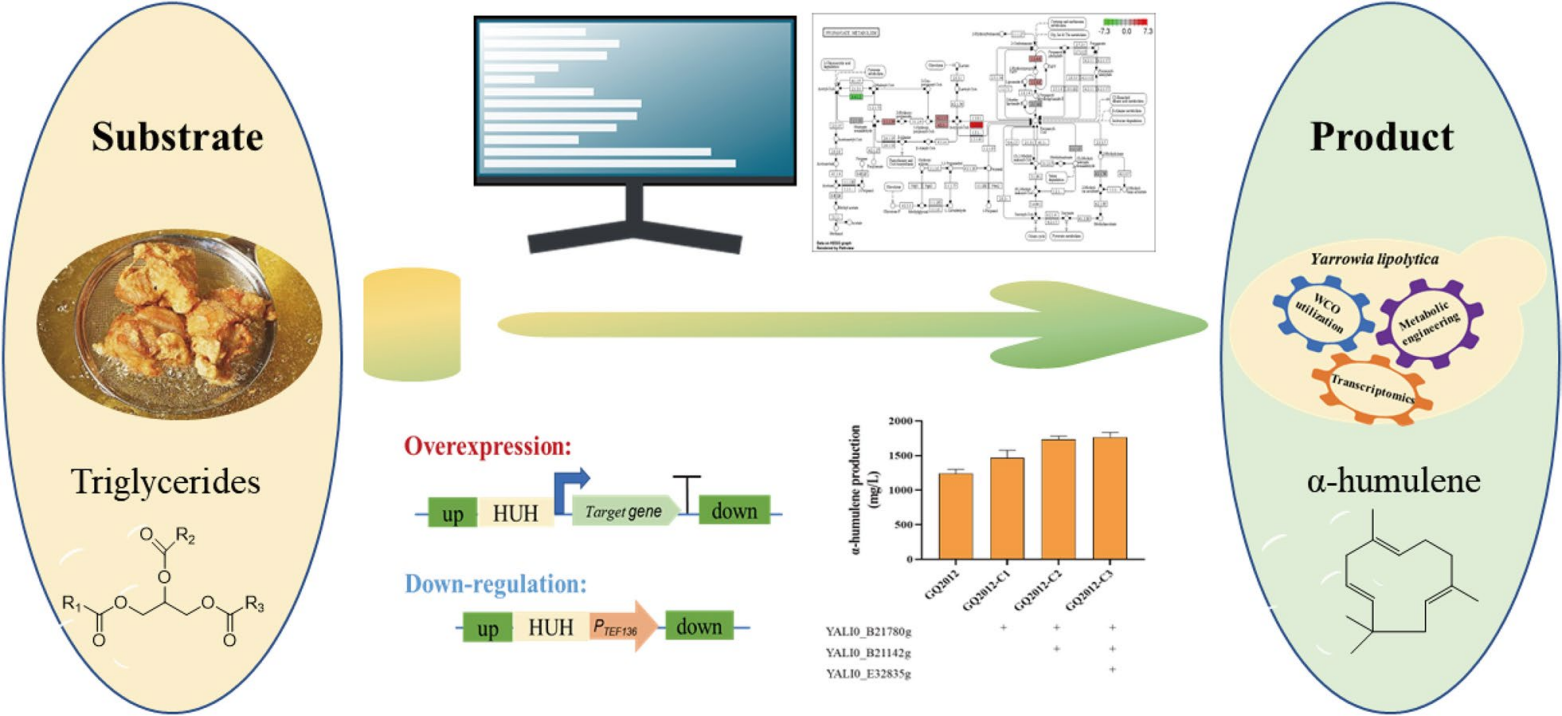

**Supplementary Information:**

The online version contains supplementary material available at 10.1186/s12934-022-01986-z.

## Introduction

α-Humulene is a sesquiterpene with potential medicinal value that can be naturally produced by the shampoo ginger plant, such as *Humulus lupulus* and *Zingiber zerumbet*, which possesses antibacterial, anti-inflammatory, and antitumor activities [1]. Although α-humulene can be obtained by plant extraction and chemical synthesis while its large-scale production is limited by high costs, low efficiency and so on [2, 3]. In recent years, with the rapid development of synthetic biology, the use of microorganisms as cell factories for the production of high-value compounds has become a growing trend [4]. Currently, heterologous production of α-humulene has been realized in a variety of hosts, including *Candida tropicalis*, *Saccharomyces cerevisiae*, and *Yarrowia lipolytica* [5–7]. Most studies have focused on metabolic engineering to modify host cells for high levels of α-humulene production. For example, Zhang et al. overexpressed the entire α-humulene synthesis pathway and the rate-limiting enzymes in *C. tropicalis*, which achieved 4115.42 mg/L α-humulene in fed-batch fermentation [5]. Furthermore, Zhang et al. and Guo et al. employed organelle engineering in *S. cerevisiae* and *Y. lipolytica* respectively, which realized a high yield of α-humulene [6, 8, 9]. However, the reduction of production expenses is also an extremely critical factor in the sustainable and industrial production of α-humulene. In order to meet the requirements of industrialization, finding renewable substitute feedstocks such as low cost or waste substrates for terpenoids production remains an area of active research.

As a kind of food industry waste, the disposal of waste cooking oil (WCO) is receiving increasing attention from various parties [10–12]. In recent years, more and more researches have been focusing on the utilization of WCO as raw and economical feedstock for the production of different value-added chemicals, which would be an attractive method from both economic and environmental protection perspectives [11, 13–15]. Notably, *Y. lipolytica* is considered an attractive industrial host suitable for conversion WCO to valuable products [16, 17]. Aside from its GRAS status, it also exhibits its robustness and good performance that can be able to grow on variable substrates [18, 19]. Up to date, *Y. lipolytica* has successfully transformed WCO to produce a variety of compounds, such as very long chain fatty acids [20], fatty acid ethyl esters [21, 22], wax esters [23] and limonene [24]. As a result, WCO is considered to be a promising and cheap carbon source while few studies were related to precise regulation of metabolic pathways and key targets. Transcriptomic analysis would provide some indications for improvement of terpenoid production.

In this study, WCO was used as the sole carbon source to produce α-humulene in *Y. lipolytica*. The combinatorial engineering strategies including omics engineering, metabolic engineering, and fermentation engineering were adopted to improve the α-humulene titer, which was expected to provide valuable knowledge on other terpenoids production in *Y. lipolytica*.

## Materials and methods

### Strains, media and culture conditions

WCO was gathered in the canteen at Nanjing Normal University. *E. coli* DH5α was used for plasmid construction and amplification and cultivated at 37 °C in a Luria–Bertani (LB) medium supplementing with 100 μg/mL ampicillin for selection. *Y. lipolytica* strains engineered in this study are based on the original *Y. lipolytica* strain GQ2012 [9]. The *Y. lipolytica* strains were cultivated at 28 °C in YPD medium (1% yeast extract, 2% peptone and 2% glucose). The YNB solid medium (0.67% yeast nitrogen base without amino acids, 1% glucose, and 1.5% agar) for selecting *Y. lipolytica* transformants was added an appropriate amount of uracil. To recycle the *URA3* selection marker, the YPD solid medium with 0.1% 5-fluoroorotic acid (5-FoA) were prepared.

### Plasmid construction and transformation

The plasmids used in this study are listed in Table 1. The differential genes used for overexpression are all endogenous and can be synthesized by polymerase chain reaction using PrimeSTAR DNA polymerase. All plasmids were constructed by ClonExpress^®^ MultiS One Step Cloning Kit (Vazyme Biotech Co., Ltd.). 1 μg of linearized plasmid was transferred into protoplast that prepared by the Zymogen Frozen EZ Yeast Transformation Kit II (Zymo Research Corporation). Subsequently, the mixture was plated on SC‐ura selection plates and incubated at 30 °C for 2–3 days to obtain positive transformations. more details were described in previous paper [8]. All strains constructed and used in this study are listed in Table 1.

**Table 1:** Main strains and plasmids used in this work

Strains and plasmids	Characteristics	Sources
*Strains*		
GQ2012	GQ2010F Δ*intE3::P*_*TEF*_*-POT1-T*_*lip1*_*, hisG-URA3-hisG*	[9]
GQ2012F	GQ2012 Δ*intE3::P*_*TEF*_*-POT1-T*_*lip1*_*, hisG*	This work
GQ2012-*YALI0_A14234g*	GQ2012F ∆s*cp2::hisG-URA3-hisG, P*_*TEFin*_*-YALI0_A14234g-T*_*XPR2*_	This work
GQ2012-*YALI0_A16379g*	GQ2012F ∆s*cp2::hisG-URA3-hisG, P*_*TEFin*_*-YALI0_ A16379g-T*_*XPR2*_	This work
GQ2012-*YALI0_D00363g*	GQ2012F ∆s*cp2::hisG-URA3-hisG, P*_*TEFin*_*-YALI0_ D00363g-T*_*XPR2*_	This work
GQ2012-*YALI0_D00671g*	GQ2012F ∆s*cp2::hisG-URA3-hisG, P*_*TEFin*_*-YALI0_ D00671g-T*_*XPR2*_	This work
GQ2012-*YALI0_D06149g*	GQ2012F ∆s*cp2::hisG-URA3-hisG, P*_*TEFin*_*-YALI0_ D06149g-T*_*XPR2*_	This work
GQ2012-*YALI0_E01210g*	GQ2012F ∆s*cp2::hisG-URA3-hisG, P*_*TEFin*_*-YALI0_ E01210g-T*_*XPR2*_	This work
GQ2012-*YALI0_E12419g*	GQ2012F ∆s*cp2::hisG-URA3-hisG, P*_*TEFin*_*-YALI0_ E12419g-T*_*XPR2*_	This work
GQ2012-*YALI0_E20471g*	GQ2012F ∆s*cp2::hisG-URA3-hisG, P*_*TEFin*_*-YALI0_ E20471g-T*_*XPR2*_	This work
GQ2012-*YALI0_E23474g*	GQ2012F ∆s*cp2::hisG-URA3-hisG, P*_*TEFin*_*-YALI0_ E23474g-T*_*XPR2*_	This work
GQ2012-*YALI0_E32835g*	GQ2012F ∆s*cp2::hisG-URA3-hisG, P*_*TEFin*_*-YALI0_ E32835g-T*_*XPR2*_	This work
GQ2012-*YALI0_F00462g*	GQ2012F ∆s*cp2::hisG-URA3-hisG, P*_*TEFin*_*-YALI0_ F00462g-T*_*XPR2*_	This work
GQ2012-*YALI0_F21923g*	GQ2012F ∆s*cp2::hisG-URA3-hisG, P*_*TEFin*_*-YALI0_ F21923g-T*_*XPR2*_	This work
GQ2012-*YALI0_F25619g*	GQ2012F ∆s*cp2::hisG-URA3-hisG, P*_*TEFin*_*-YALI0_ F25619g-T*_*XPR2*_	This work
GQ2012-*YALI0_F27005g*	GQ2012F ∆s*cp2::hisG-URA3-hisG, P*_*TEFin*_*-YALI0_ F27005g-T*_*XPR2*_	This work
GQ2012-*YALI0_B16170g*	GQ2012F Δ*P*_*YALI0_B16170g*_:: *hisG-URA3-hisG*, *P*_*TEF136*_*,*	This work
GQ2012-*YALI0_B21142g*	GQ2012F Δ*P*_*YALI0_B21142g*_:: *hisG-URA3-hisG*, *P*_*TEF136*_	This work
GQ2012-*YALI0_B21780g *(GQ2012-C1)	GQ2012F Δ*P*_*YALI0_B21780g*_:: *hisG-URA3-hisG*, *P*_*TEF136*_	This work
GQ2012-*YALI0_C00803g*	GQ2012F Δ*P*_*YALI0_C00803g*_:: *hisG-URA3-hisG*, *P*_*TEF136*_	This work
GQ2012-*YALI0_C05951g*	GQ2012F Δ*P*_*YALI0_C05951g*_:: *hisG-URA3-hisG*, *P*_*TEF136*_	This work
GQ2012-*YALI0_C10311g*	GQ2012F Δ*P*_*YALI0_C10311g*_:: *hisG-URA3-hisG*, *P*_*TEF136*_	This work
GQ2012-*YALI0_E11561g*	GQ2012F Δ*P*_*YALI0_E11561g*_:: *hisG-URA3-hisG*, *P*_*TEF136*_	This work
GQ2012-*YALI0_E12947g*	GQ2012F Δ*P*_*YALI0_E12947g*_:: *hisG-URA3-hisG*, *P*_*TEF136*_	This work
GQ2012-*YALI0_F13937g*	GQ2012F Δ*P*_*YALI0_F13937g*_:: *hisG-URA3-hisG*, *P*_*TEF136*_	This work
GQ2012-*YALI0_F27709g*	GQ2012F Δ*P*_*YALI0_F27709g*_:: *hisG-URA3-hisG*, *P*_*TEF136*_	This work
GQ2012-C1F	GQ2012-C1 Δ*P*_*YALI0_B21780g*_:: *hisG*, *P*_*TEF136*_	This work
GQ2012-C2	GQ2012-C1F Δ*P*_*YALI0_B21142g*_:: *hisG-URA3-hisG*, *P*_*TEF136*_	This work
GQ2012-C2F	GQ2012-C2 Δ*P*_*YALI0_B21142g*_:: *hisG*, *P*_*TEF136*_	This work
GQ2012-C3	GQ2012-C2F ∆s*cp2::hisG-URA3-hisG, P*_*TEFin*_*-YALI0_ E32835g-T*_*XPR2*_	This work
*Plasmids*		
pUC-HUH-scp2-*YALI0_A14234g*	*hisG-URA3-hisG, P*_*TEFin*_*-YALI0_A14234g-T*_*XPR2*_*,* cassette in pUC-HUH-scp2	This work
pUC-HUH-scp2-*YALI0_A16379g*	*hisG-URA3-hisG, P*_*TEFin*_*-YALI0_A16379g-T*_*XPR2*_*,* cassette in pUC-HUH-scp2	This work
pUC-HUH-scp2-*YALI0_D00363g*	*hisG-URA3-hisG, P*_*TEFin*_*-YALI0_D00363g-T*_*XPR2*_*,* cassette in pUC-HUH-scp2	This work
pUC-HUH-scp2-*YALI0_D00671g*	*hisG-URA3-hisG, P*_*TEFin*_*-YALI0_D00671g-T*_*XPR2*_*,* cassette in pUC-HUH-scp2	This work
pUC-HUH-scp2-*YALI0_D06149g*	*hisG-URA3-hisG, P*_*TEFin*_*-YALI0_D06149g-T*_*XPR2*_*,* cassette in pUC-HUH-scp2	This work
pUC-HUH-scp2-*YALI0_E01210g*	*hisG-URA3-hisG, P*_*TEFin*_*-YALI0_E01210g-T*_*XPR2*_*,* cassette in pUC-HUH-scp2	This work
pUC-HUH-scp2-*YALI0_E12419g*	*hisG-URA3-hisG, P*_*TEFin*_*-YALI0_E12419g-T*_*XPR2*_*,* cassette in pUC-HUH-scp2	This work
pUC-HUH-scp2-*YALI0_E20471g*	*hisG-URA3-hisG, P*_*TEFin*_*-YALI0_E20471g-T*_*XPR2*_*,* cassette in pUC-HUH-scp2	This work
pUC-HUH-scp2-*YALI0_E23474g*	*hisG-URA3-hisG, P*_*TEFin*_*-YALI0_E23474g-T*_*XPR2*_*,* cassette in pUC-HUH-scp2	This work
pUC-HUH-scp2-*YALI0_E32835g*	*hisG-URA3-hisG, P*_*TEFin*_*-YALI0_E32835g-T*_*XPR2*_*,* cassette in pUC-HUH-scp2	This work
pUC-HUH-scp2-*YALI0_F00462g*	*hisG-URA3-hisG, P*_*TEFin*_*-YALI0_F00462g-T*_*XPR2*_*,* cassette in pUC-HUH-scp2	This work
pUC-HUH-scp2-*YALI0_F21923g*	*hisG-URA3-hisG, P*_*TEFin*_*-YALI0_F21923g-T*_*XPR2*_*,* cassette in pUC-HUH-scp2	This work
pUC-HUH-scp2-*YALI0_F25619g*	*hisG-URA3-hisG, P*_*TEFin*_*-YALI0_F25619g-T*_*XPR2*_*,* cassette in pUC-HUH-scp2	This work
pUC-HUH-scp2-*YALI0_F27005g*	*hisG-URA3-hisG, P*_*TEFin*_*-YALI0_F27005g-T*_*XPR2*_*,* cassette in pUC-HUH-scp2	This work
pUC-HUH-P_TEF136_-*YALI0_B16170g*	replace the native promoter of the *YALI0_B16170g* with P_TEF136_	This work
pUC-HUH-P_TEF136_-*YALI0_B21142g*	replace the native promoter of the *YALI0_B21142g* with P_TEF136_	This work
pUC-HUH-P_TEF136_-*YALI0_B21780g*	replace the native promoter of the *YALI0_B21780g* with P_TEF136_	This work
pUC-HUH-P_TEF136_-*YALI0_C00803g*	replace the native promoter of the *YALI0_C00803g* with P_TEF136_	This work
pUC-HUH-P_TEF136_-*YALI0_C05951g*	replace the native promoter of the *YALI0_C05951g* with P_TEF136_	This work
pUC-HUH-P_TEF136_-*YALI0_C10311g*	replace the native promoter of the *YALI0_C10311g* with P_TEF136_	This work
pUC-HUH-P_TEF136_-*YALI0_E11561g*	replace the native promoter of the *YALI0_E11561g* with P_TEF136_	This work
pUC-HUH-P_TEF136_-*YALI0_E12947g*	replace the native promoter of the *YALI0_E12947g* with P_TEF136_	This work
pUC-HUH-P_TEF136_-*YALI0_F13937g*	replace the native promoter of the *YALI0_F13937g* with P_TEF136_	This work
pUC-HUH-P_TEF136_-*YALI0_F27709g*	replace the native promoter of the *YALI0_F27709g* with P_TEF136_	This work

### Analysis of α-humulene content

Analytical methods of α-humulene content were described previously [25].

### Transcriptome analysis

*Y. lipolytica* GQ2012 was cultured at 30 °C with YPD medium and WCO medium, respectively. After 48 h of culture, centrifugation was performed at 4000 rpm for 5 min and the supernatant was discarded. Next, the cells were resuspended with normal saline and centrifuged for 10 min, then the cells were quickly put into liquid nitrogen for freezing. Three backup samples were prepared for each yeast sample. RNA extraction and sequencing were carried out in Beijing Novogene Bioinformatics Technology Co., Ltd.

### Bioreactor fermentations

For the shake-flask fermentations, 40 mL WCO medium containing 1% yeast extract, 2% peptone and WCO was cultured for 4 days at 30 °C, 220 rpm in a 250 mL flask. The seed solution was added to the WCO medium after overnight cultivation, and the initial OD600 was controlled at 0.1.

For the fed-batch fermentations, 2 L WCO medium containing 150 mL WCO, 20 g/L yeast extract and 40 g/L peptone were added into 5 L parallel-bioreactor (T&J Bio-engineering Co., Ltd, Shanghai, China). The 200 mL seed culture in a shake flask was transferred into the bioreactor and the initial OD_600_ was set to 0.5 rel. AU. The temperature, agitation speed and dissolved oxygen were maintained at 28 °C, 500 rpm and 20% of air saturation, respectively. The α-humulene titer were measured every 12 h. The pH was maintained at 5.5 regulated by NaOH or HCl. The fed-batch process was initiated after a 36 h cultivation at a rate of 8 mL/h from an WCO solution.

## Results and discussion

### Feasibility of using WCO as feedstock for α-humulene production

*Y. lipolytica* can directly utilize triacylglycerols (TAGs) as a sole carbon source which are hydrolyzed to fatty acids (FAs) and glycerol. The free FAs, with the help of an active transport system, are transferred into the fungal cells. Inside the cells, the free FAs are either biotransformed for the synthesis of new components (microbial lipids) of the cells or degraded by the strong β-oxidation process [26, 27]. As a result, large amounts of acetyl-CoA were produced, which can be used not only for cell growth, but also for the biosynthesis of other high-value compounds including terpenoids [24]. In our previous study, we obtained a peroxisome-engineering *Y. lipolytica* GQ2012 that localized 15 genes to peroxisomes, including the whole α-humulene biosynthetic pathway, additional double copies of α-humulene synthase (*ACHS2*) and hydroxymethylglutaryl-CoA reductase (*HMG1*), as well as single copies of 3-ketoacyl-CoA thiolase (*POT1*) and peroxisomal adenine nucleotide transporter (*ANT1*). In total 3.2 g/L of α-humulene was obtained in the best-performing strain GQ2012, which was the highest titer reported at that time [9]. On the basis of this, to further reduce the production cost, WCO was used as the sole carbon source for the production of α-humulene.

**Fig. 1:**
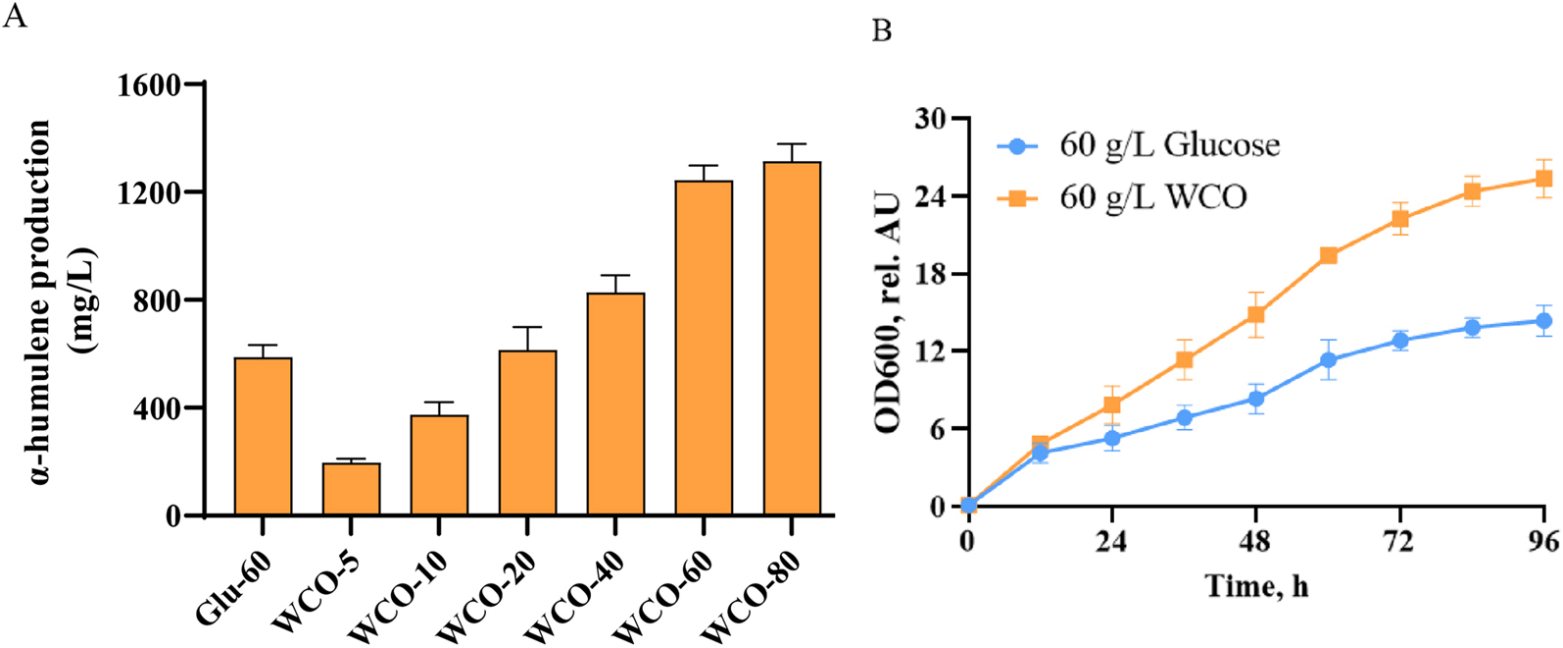
Feasibility of producing α-humulene with WCO as the sole carbon source. **A** Influence of different WCO concentrations on α-humulene production. **B** Differences in growth between the GLU group and the WCO group. The data represent the means ± standard deviations (n = 3)

As shown in Fig. 1a, different concentrations of WCO (5, 10, 20, 40, 60, 80 g/L) were investigated and 60 g/L glucose was used as a control. The results showed that the strain GQ2012 can be able to produce α-humulene over the range of WCO concentrations examined. Compared to the 587 mg/L α-humulene produced by the control strain, the α-humulene produced by GQ2012 using WCO as the carbon source enhanced with the increasing concentration from 196.7 to 1313.3 mg/L. We found that when the WCO concentration was higher than 20 g/L, the yield of α-humulene was higher than that of the control group. In addition, the titer of α-humulene increased slowly when the WCO concentration increased from 60 to 80 g/L. After comprehensive consideration, 60 g/L WCO was chosen for subsequent experiments, which can obtain 1243.3 mg/L α-humulene. It worth mentioning that *Y. lipolytica* appeared to prefer WCO over glucose as a source of carbon and energy. The growth rates of strain GQ2012 using 60 g/L WCO are higher than those using 60 g/L glucose as carbon source (Fig. 1b), which indicated that *Y. lipolytica* can rapidly utilize WCO for cell growth and α-humulene biosynthesis. In addition, we also tried using WCO from different canteens as feedstock to produce α-humulene which showed that WCO from different sources had basically no influence on the yield of α-humulene (Additional file 1: Fig. S1). Taken together, we believe that WCO potentially serve as a platform for sustainable and economical production of α-humulene.

### Transcriptomics analysis of the *Y. lipolytica* GQ2012 with different carbons

**Fig. 2:**
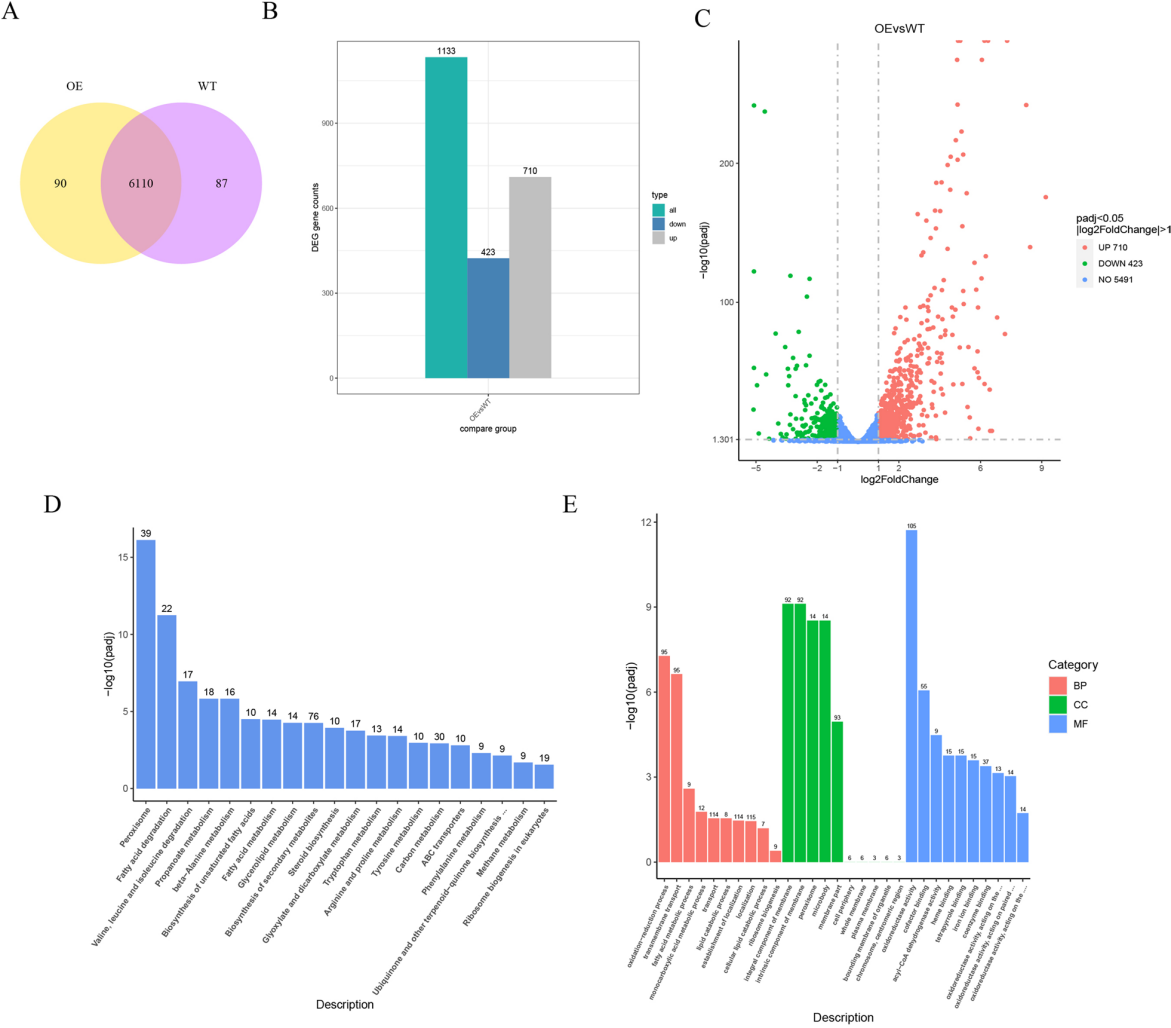
Transcriptomic analysis and annotation of DEGs between the GLU group and the WCO group. **A** Venn diagram. **B** DEGs counts. **C** Volcano plots. **D** KEGG annotation of the DEGs. **E** GO annotation of the DEGs. the WCO group (OE); the GLU group (WT); Biological processes (BP); Cellular components (CC); Molecular functions (MF)

To further enhance α-humulene production in *Y. lipolytica*, transcriptome analysis was performed to study the differences of global gene expression between glucose and WCO as the only carbon source. Three biological replicates were carried out for each strain to ensure the reproducibility. First of all, in order to determine whether the differential genes of the two carbon source fermentation strains are related, Venn diagram analysis was performed on their significantly differential expression genes. As shown in Fig. 2a, the WCO group and GLU group have 90 and 87 uniquely expressed genes respectively. In addition, according to the criteria of DESeq2 padj ≤ 0.05 and |log2FoldChange| ≥ 1.0 for screening differences, the number of differential genes (both up- and down-regulated) was counted in Fig. 2b. The volcano plot can visually display the differential gene distribution of each comparison combination (Fig. 2c). The result showed that a total of 710 differentially expressed genes (DEGs) were upregulated and 423 DEGs were downregulated.

Subsequently, Kyoto Encyclopedia of Genes and Genomes (KEGG) annotation was conducted to study the intersection of the significantly different expressed genes in the two different carbon sources. As shown in Fig. 2d, it was found that these differentially expressed genes were mainly involved in peroxisomes, fatty acid degradation and valine, leucine and isoleucine degradation, etc. The highest numbers of DEGs related to peroxisomes was observed. To the best of our knowledge, peroxisomes, as one of the organelles, play a key role in redox signalling and lipid homeostasis. They contribute to many crucial metabolic processes such as fatty acid oxidation, biosynthesis of ether lipids and free radical detoxification, which was consistent with the utilization of WCO as the sole carbon source for the α-humulene production in the peroxisome-engineering strain GQ2012. When the released FAs incorporated inside the yeast cells through a process that is not random and will either be metabolized through the β-oxidation pathway, which used for cell growth and metabolite production. The expression level of most genes in peroxisomes was significantly up-regulated, which indicated that the use of WCO facilitated this process. It is worth mentioning that the expression level of acyl-CoA oxidase (YALI0E32835g) was most significantly up-regulated, which plays an important role in the relevant pathways such as fatty acid degradation, beta-Alanine metabolism, alpha-Linolenic acid metabolism and so on. Besides, fatty acid metabolism mainly involves the production of acetyl CoA that is the universal precursor for terpenoid biosynthesis. As a result, this also explained the importance and necessity of fatty acid metabolism in α-humulene biosynthesis.

In addition, Gene Ontology (GO) annotation and classification analysis were conducted to show the potential functions of the DEGs treated by WCO. As shown in Fig. 2e, DEGs are divided into three main categories based on the different functions, including biological processes (BP), cellular components (CC) and molecular functions (MF). Oxidation–reduction process, integral and intrinsic component of membrane, and oxidoreductase activity were the most abundant annotation terms in each of the three GO categories. It is worth mentioning that we found that a large of number of DEGs were related to the categories of localization, establishment of localization and transport, which may be related to the localization of the α-humulene biosynthetic pathway to peroxisomes and the utilization and degradation of WCO. Overall, the results of the transcriptomic analysis are consistent with the use of WCO to improve α-humulene production in the peroxisome-engineering strain. We speculated that these apparent changes are caused by differences in the expression levels of genes associated with localization and transport.

### Enhancing α-humulene production by exploring potential targets

**Fig. 3:**
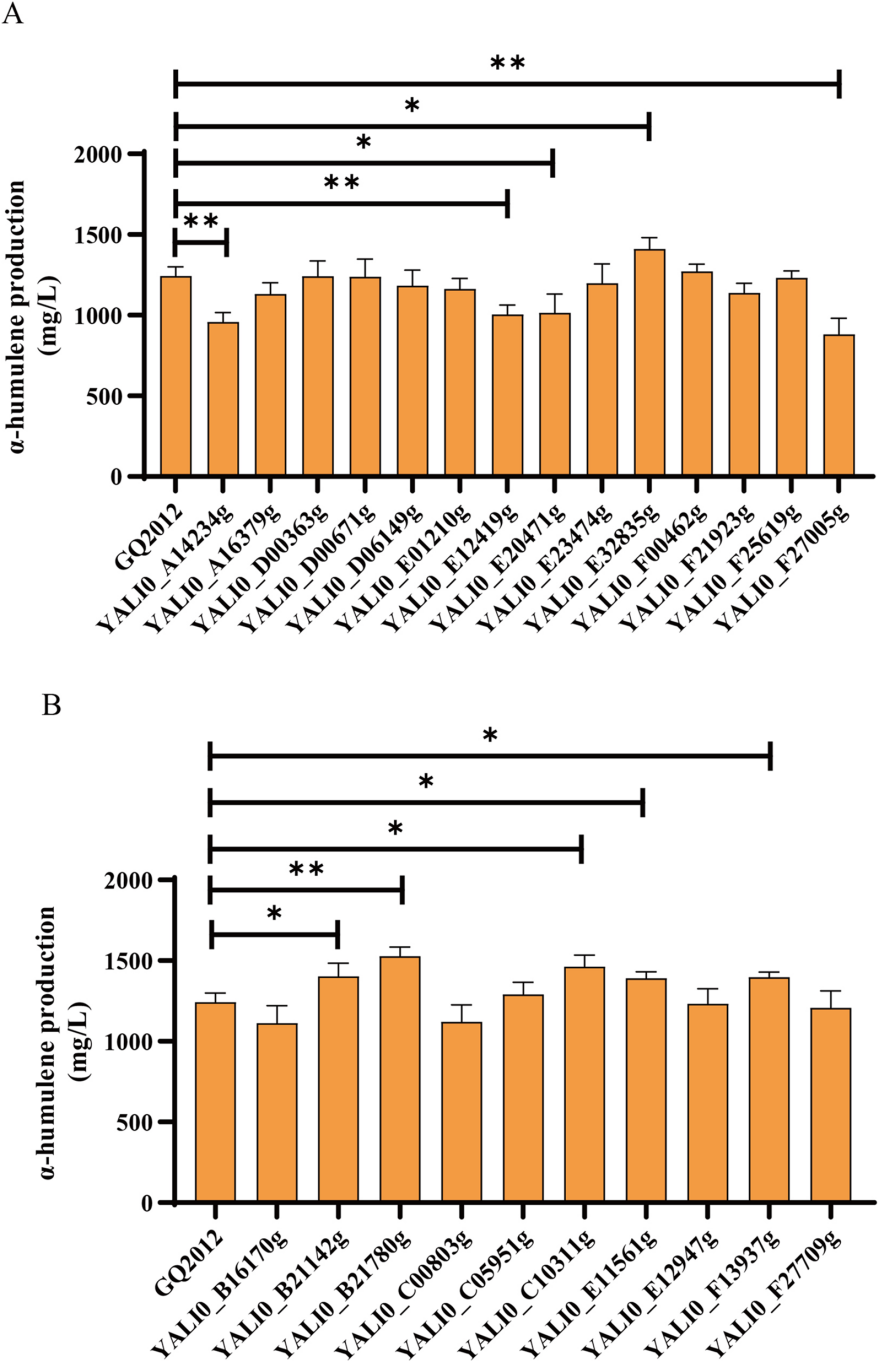
Influence of differential genes regulation on α-humulene production. **A** Overexpression of genes with significantly up-regulated transcript levels. **B** down-regulation of gene with significantly down-regulated transcript levels. The data represent the means ± standard deviations (n = 3)

To further search for potential target genes involved in the conversion of WCO to α-humulene production, fourteen significantly up-regulated genes that log2 FoldChange > 6, were overexpressed individually and investigated for the influences on α-humulene overproduction. To this end, fourteen single-gene overexpression strains were constructed on the basis of the strain GQ2012. All the genes were controlled under the strong promoter P_TEFin_ and integrated into genome loci. As shown in Fig. 3a and Additional file 1: Fig. S2A, overexpression of YALI0_E32835g promoted the production of α-humulene with the titer of 1410.12 mg/L, which was 13.4% higher than the control strain, while other genes had little or negative effects on α-humulene production. Looking deeper into the reason, we found that YALI0_E32835g is acyl-CoA oxidase with the molecular function of oxidoreductase. The gene is involved in a variety of biological processes including fatty acid degradation, peroxisome process, α-linolenic acid metabolism and so on, which revealed that this gene plays a pivotal role in the production of α-humulene in the peroxisomes.

Similarly, those genes which were significantly down-regulated were also examined for their effects on α-humulene titer. Ten genes that log2FoldChange > 6, were chosen to down-regulate their transcript expression levels. The weak P_TEF136_ promoter was used to replace the endogenous promoter respectively. As shown in Fig. 3b and Additional file 1: Fig. S2B, the results showed that down-regulation of the expression levels of seven genes improved α-humulene production. Among these strains, down-regulation of YALI0_B21780g expression improved α-humulene production most significantly, which reached up to 22.8% higher than the control strain GQ2012, reaching to 1526.7 mg/L. We found that the function of YALI0_B21780g is similar to glycerol 2-dehydrogenase (NADP(+)) *GCY1* from *S. cerevisiae* S288C that involved in glycerolipid metabolism. This protein is involved in glycerol catabolism under microaerobic conditions that consumes large amounts of NADP(+). It is worth mentioning that the process of fatty acid degradation also requires the participation of NADP(+). It is possible that the down-regulation of YALI0_B21780g weakened the competition for NADP(+), which facilitated the continuous catabolism of WCO, thus promoting the production of α-humulene. In addition, regulation of YALI0_C10311g also showed a good property for promoting α-humulene production, resulting in a 17.7% increase in yield to 1463.3 mg/L. We found that YALI0_C10311g is a potassium (K) uptake protein in KUP system involved in the process of ion transport with transmembrane transporter activity. The KUP system is more extensively studied in *Escherichia coli* [28, 29]. The KUP system is more active at lower pH values for maintaining cellular homeostasis. The apparent down-regulation of the transcript level of YALI0_C10311g may be due to the lesser K demand in maintaining a steady-state environment, thus making the KUP system less important. In addition to the two genes mentioned above, four genes including YALI0_B21142g, YALI0_C05951g, YALI0_E11561g and YALI0_F13937g also improved the yield of α-humulene, but not significantly while other genes had an adverse effect on α-humulene production.

Overall, we found that precise regulation of genes that are significantly up- or down-regulated can help identify some potential targets, which is essential for effective improvement of α-humulene biosynthesis. However, we found that engineering a single gene had a marginal influence on α-humulene production, which suggested that central metabolism should be systematically rewired for WCO biotransformation.

### Enhancing α-humulene production by metabolic rewiring

**Fig. 4:**
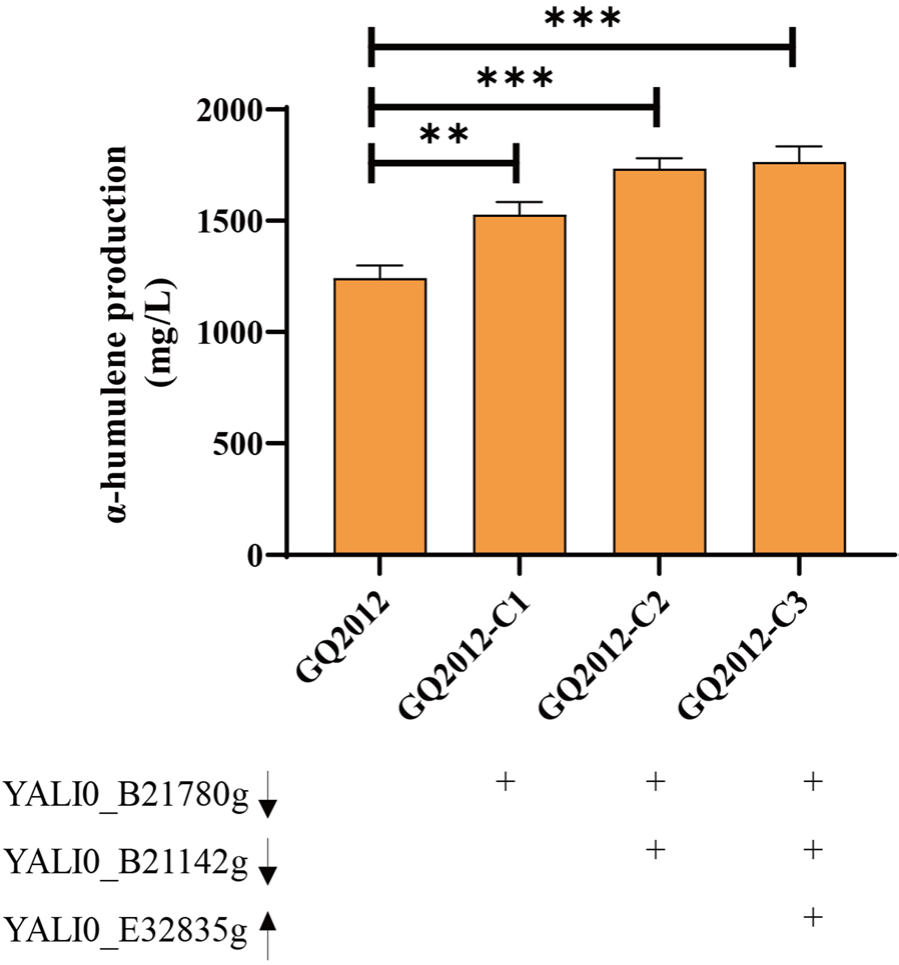
Rational metabolic engineering to improve α-humulene production. YALI0_B21780g and YALI0_B21142g were down-regulated. YALI0_E32835g was overexpressed. The data represent the means ± standard deviations (n = 3)

Based on the above experimental results, overexpression or knockdown of some differential genes is beneficial to improve α-humulene production. As a result, three genes including YALI0_B21780g, YALI0_C10311g and YALI0_E32835g, were selected to investigate the potential synergistic effects on α-humulene production. As shown in Fig. 4 and Additional file 1: Fig. S3, the promoter of YALI0_C10311g was replaced with P_TEF136_ to obtain strain GQ2012-C2 on the basis of strain GQ2012-C1. We found that it is significantly increased by 13.5% in the level of α-humulene which indicated that combinatorial regulation is an effective strategy to upgrade the α-humulene level. Encouraged by the above experimental results, we further introduced YALI0_E32835g into strain GQ2012-C2 to obtain strain GQ2012-C3, which obtained 1763.33 mg/L α-humulene. However, this titer was not significantly higher as we expected, but only 30 mg/L higher than the strain of GQ2012-C2. In general, the results showed that all the engineered strains showed greater α-humulene production than the initial strain GQ2012.

### Fed-batch fermentation in a 5-L bioreactor

**Fig. 5:**
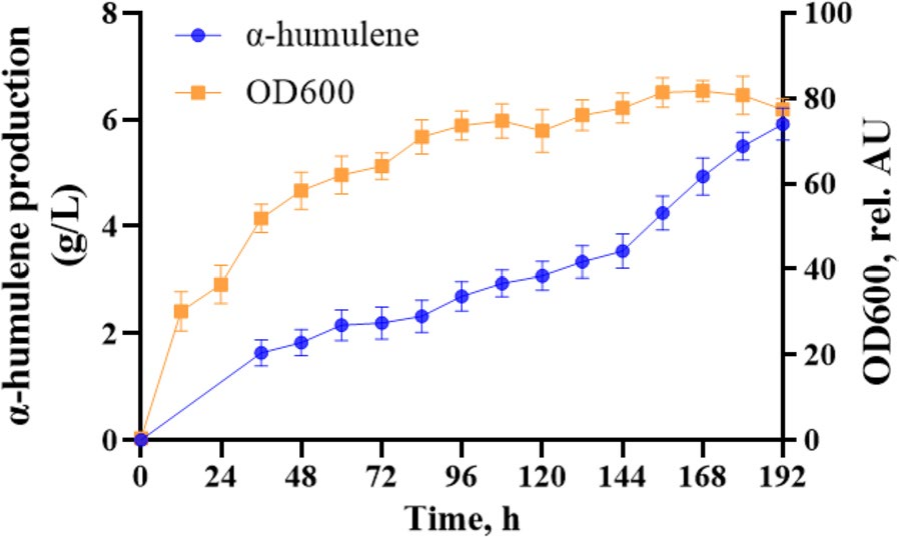
Fed‐batch bioreactor fermentation of the GQ2012-C3 strain for α‐humulene production. The data represent the means ± standard deviations (n = 3)

Finally, the best engineered strain was evaluated in 5-L bioreactor for high-level α-humulene production with WCO as sole carbon source. As shown in Fig. 5, we found that the α-humulene titer increased slowly until 144 h and increased rapidly when the addition of WCO to the reactor is stopped. Finally, the highest α-humulene titer was obtained at 192 h that was 5.9 g/L. In addition, we found that the biomass of strain GQ2012-C3 increased rapidly in the first 72 h, especially in the first 36 h. In the later stage of fermentation process, the biomass growth increased at a slow rate. Eventually, the biomass of strain GQ2012-C3 reached to 77 at 192 h. The results demonstrated the ability of the engineered *Y. lipolytica* to transform WCO. The high yields demonstrated the potential of engineered strains obtained in this study to transform WCO into high-value chemicals. Our study also demonstrated that the circular bioeconomy concept can be an effective model for scale-up production of valuable biochemical, in particular with the valorization of WCO as raw material.

## Conclusions

In this study, we combined omics engineering, metabolic engineering, and fermentation engineering to enhance the potential of *Y. lipolytica* to produce α-humulene using WCO. A feasible strategy was used to reduce production costs and enhance the α-humulene productivity of microbial cell factories. In summary, the results showed that our engineered *Y.* *lipolytica* can serve as a platform strain for the production of valuable chemicals while laying the foundation for more future applications of WCO bioconversion in *Y. lipolytica*.

## Supplementary Information

**Additional file 1: Fig. S1.** Influence of WCO from different canteens on α-humulene production. **Fig. S2.** Influence of differential genes regulation on α-humulene production (DCW). (A) Overexpression of genes with significantly up-regulated transcript levels. (B) down-regulation of gene with significantly down-regulated transcript levels. The data represent the means ± standard deviations (n = 3). **Fig. S3.** Rational metabolic engineering to improve α-humulene production (DCW). YALI0_B21780g and YALI0_B21142g were down-regulated. YALI0_E32835g was overexpressed. The data represent the means ± standard deviations (n = 3).
