# Supplementary material for: High-yield α-humulene production in Yarrowia lipolytica from waste cooking oil based on transcriptome analysis and metabolic engineering
Source: Microb Cell Fact. 2022 Dec 24;21:271. doi: 10.1186/s12934-022-01986-z (PMC9790127; doi:10.1186/s12934-022-01986-z)
Supplement: Supplementary file 1 — Additional file 1: Fig. S1. Influence of WCO from different canteens on α-humulene production. Fig. S2. Influence of differential genes regulation on α-humulene production (DCW). (A) Overexpression of genes with significantly up-regulated transcript levels. (B) down-regulation of gene with significantly down-regulated transcript levels. The data represent the means ± standard deviations (n = 3). Fig. S3. Rational metabolic engineering to improve α-humulene production (DCW). YALI0_B21780g and YALI0_B21142g were down-regulated. YALI0_E32835g was overexpressed. The data represent the means ± standard deviations (n = 3). [file 12934_2022_1986_MOESM1_ESM.docx]

High-yield α-humulene production in *Yarrowia lipolytica* from waste cooking oil based on transcriptome analysis and metabolic engineering

Qi Guo^a^, Qian-Qian Peng^b^, Ying-Ying Chen^b^, Ping Song^b^, Xiao-Jun Ji^a^, He Huang^a,b,c*^, Tian-Qiong Shi^b*^

^a^ College of Biotechnology and Pharmaceutical Engineering, Nanjing Tech University, No. 30 South Puzhu Road, Nanjing 211816, People’s Republic of China

^b^ School of Food Science and Pharmaceutical Engineering, Nanjing Normal University, 2 Xuelin Road, Qixia District, Nanjing 210046, People’s Republic of China

^c^ College of Pharmaceutical Sciences, Nanjing Tech University, No. 30 South Puzhu Road, Nanjing 211816, People’s Republic of China

*Corresponding Author: College of Biotechnology and Pharmaceutical Engineering, Nanjing Tech University (Tian-Qiong Shi, [tqshi@njnu.edu.cn](mailto:tqshi@njnu.edu.cn); He Huang, [huangh@njnu.edu.cn](mailto:huangh@njnu.edu.cn))


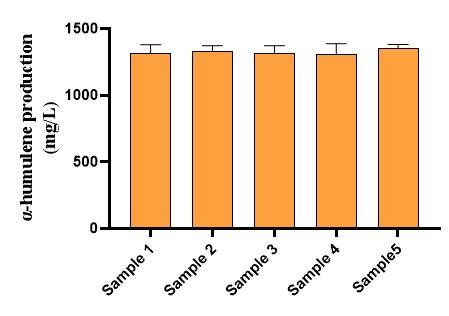


**Fig. S1.** Influence of WCO from different canteens on α-humulene production.


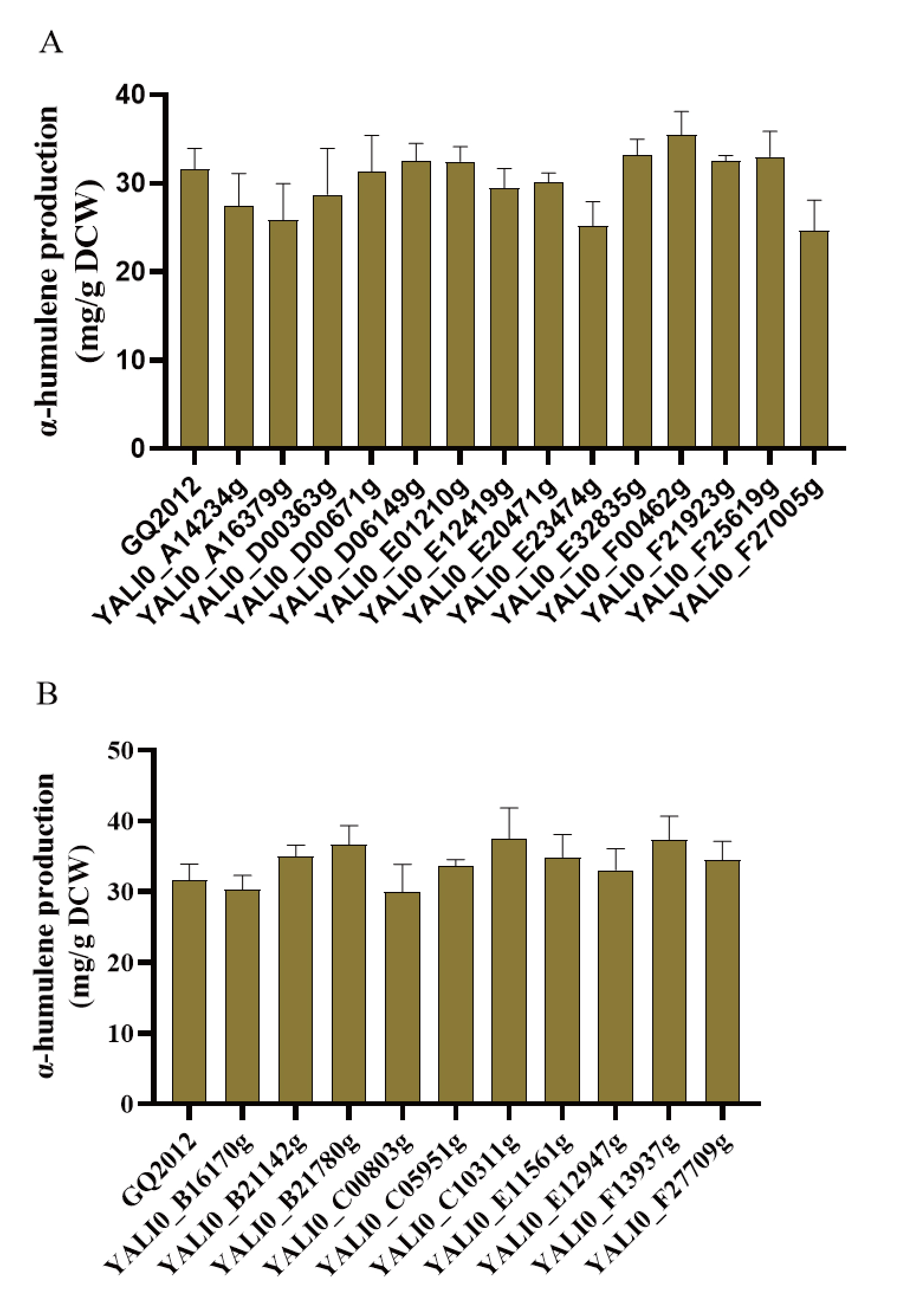


**Fig. S2.** Influence of differential genes regulation on α-humulene production (DCW). (A) Overexpression of genes with significantly up-regulated transcript levels. (B) down-regulation of gene with significantly down-regulated transcript levels. The data represent the means ± standard deviations (n = 3).


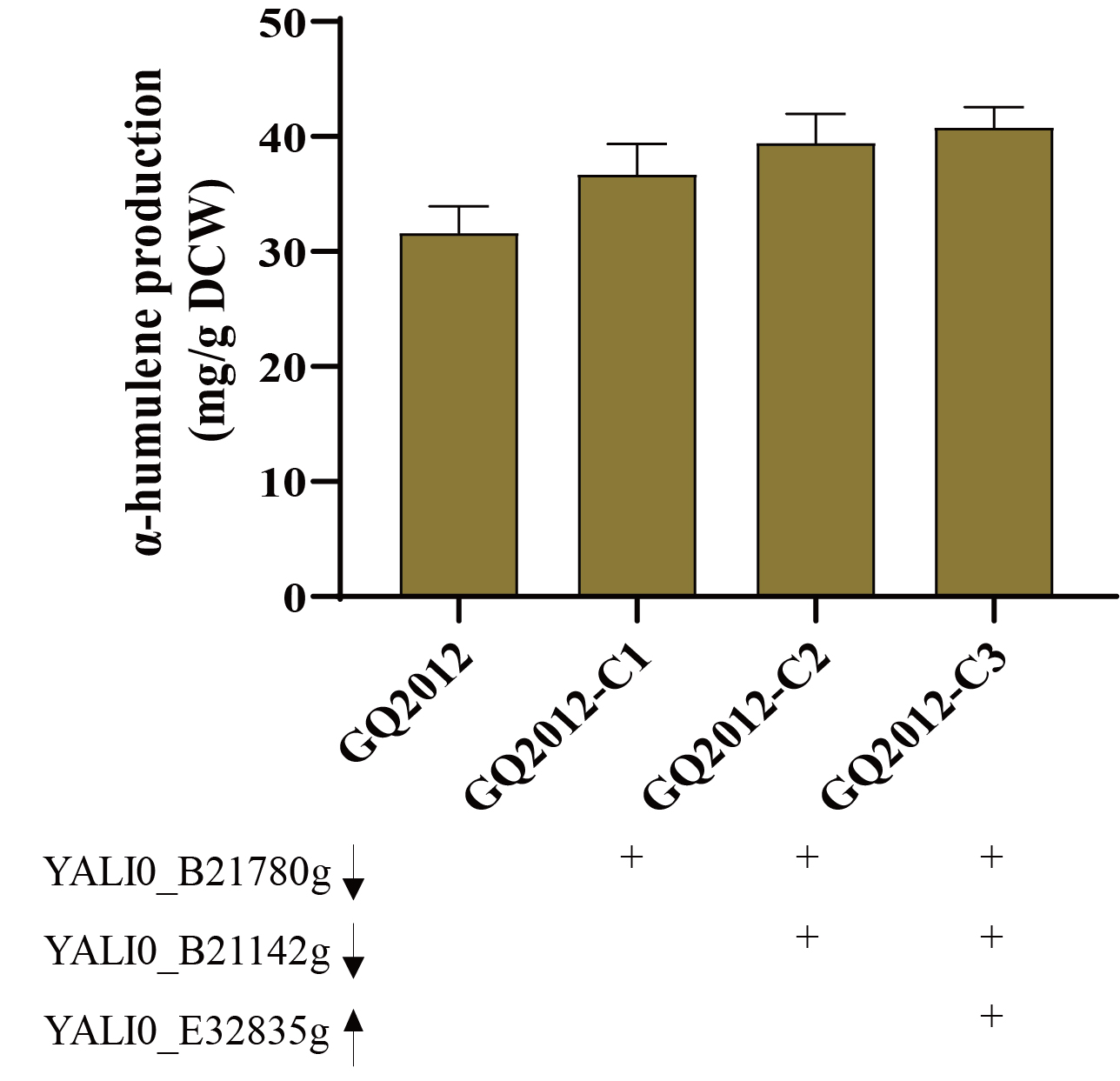


**Fig. S3.** Rational metabolic engineering to improve α-humulene production (DCW). YALI0_B21780g and YALI0_B21142g were down-regulated. YALI0_E32835g was overexpressed. The data represent the means ± standard deviations (n = 3).
